# Inversion of Central Venous Ports in Children Under Six Years Old: A Retrospective Analysis of 154 Oncology Patients

**DOI:** 10.7759/cureus.63106

**Published:** 2024-06-25

**Authors:** Yuji Koretsune, Shunsuke Sugawara, Miyuki Sone, Hiroki Higashihara, Ayumu Arakawa, Chitose Ogawa, Masahiko Kusumoto, Noriyuki Tomiyama

**Affiliations:** 1 Diagnostic and Interventional Radiology, Osaka University, Osaka, JPN; 2 Diagnostic Radiology, National Cancer Center Hospital, Tokyo, JPN; 3 High Precision Image-Guided Percutaneous Intervention, Osaka University Hospital, Osaka, JPN; 4 Pediatrics, National Cancer Center Hospital, Tokyo, JPN; 5 Pediatric Oncology, National Cancer Center Hospital, Tokyo, JPN

**Keywords:** risk factor, infectious complication, mechanical complication, inversion rate, children, central venous port

## Abstract

Background

Although some reports have evaluated the safety and efficacy of central venous port (CVP) placement in pediatric patients, the data about the inversion rate of the device and its risk factors are scarce. Therefore, this study aimed to evaluate the inversion rates of CVPs and their associated risk factors in pediatric patients.

Methodology

Between January 2010 and December 2021, 154 consecutive children (75 boys; median age, 28.5 months; range, 2-71 months) who underwent CVP placement at our center were included in this study. The primary outcome was the CVP inversion rate, and the secondary outcomes included technical success rate, intraoperative complications, and infectious complications. Intraoperative complications were evaluated according to the Society of Interventional Radiology guidelines. Patients under two years old were classified as the younger group and those aged ≥two years as the older group.

Results

The CVP inversion rate was 4.6% (n = 7/153), equivalent to 0.08 × 1,000 catheter-days. The inversion rate was significantly higher in the younger group (under two years old, 11.2%) than in the older group (≥two years old, 1.0%) according to the univariate analysis (p = 0.00576). The technical success rate was 99.4% (n = 153/154), and mild adverse events were observed during the procedure in three (1.9%) patients. Infectious complications were observed in 16 (10.5%) patients, equivalent to 0.19 × 1,000 catheter-days.

Conclusions

The CVP inversion rate was significantly higher in younger children (under two years old) than in older children (≥two years old).

## Introduction

The central venous port (CVP) plays an integral role in the care of pediatric patients by facilitating the administration of chemotherapeutic drugs, blood products, other medications, and total parenteral nutrition. Since Morris et al. first reported radiology-guided CVP placement in 1992 [1], its safety and efficacy have been well-established in adult populations [2-8]. Although the utility of CVP implantation in pediatric populations has been evaluated, relatively fewer reports are available in this regard [9-11]. To ensure safe and reliable central venous access, it is crucial to mitigate the complications that may interrupt continuous CVP use, such as mechanical and infectious complications. Although previous studies have evaluated the frequency and associated risk factors of infection-related complications in pediatric patients [12-15], limited data are available on the incidence of mechanical complications occurring after CVP insertion in this patient demographic [16]. Inversion of the device in the pocket is one of the major mechanical complications that necessitate an incision and replacement of the device once it occurs. Although a previous study established that certain rectangular port designs with narrow bases are potential risk factors for CVP inversion [17], the study was conducted in adult populations only. We hypothesized that younger children would have an increased risk of port inversion owing to their higher rates of movements involving the shoulder joints (e.g., crawling). Therefore, this study aimed to compare CVP inversion rates and explore their associated risk factors in pediatric patients retrospectively.

## Materials and methods

This single-institution retrospective analysis was performed in accordance with the tenets of the Declaration of Helsinki and was approved by the institution’s ethics committee (approval number: 2022-110). The requirement for informed consent was waived owing to the retrospective nature of the study. Written informed consent for all procedures was obtained from all of the patients’ guardians.

Procedures

**Figure 1:**
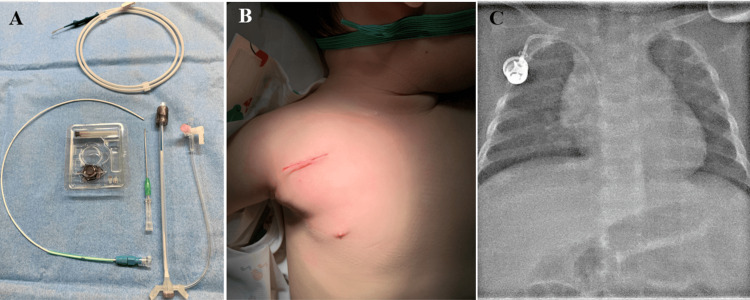
Procedural details. (A) Photograph of the CELSITE small port, 5 Fr Anthron PU catheter, peel-away sheath, 18 G needle, and 0.035” wire. (B) Chest images after the central venous port implantation in a two-year-old patient. (C) Chest radiography following the procedure.

All procedures were performed by experienced operators in an interventional radiology suite (Figure 1). Conscious sedation was administered to all patients via intravenous injection by trained pediatricians and consisted of thiamylal sodium (Titosol; Kyorin Pharmaceutical, Tokyo, Japan) at an initial dose of 3 mg/kg, with an additional dose of 1 mg/kg given if a patient exhibited agitation or discomfort. Additionally, ketamine hydrochloride (Ketalar; Daiichi Sankyo, Tokyo, Japan) was also administered intravenously to some patients at the discretion of the attending pediatric physician. None of the patients received general anesthesia. Antibiotic prophylaxis was also administered at the discretion of the attending pediatrician.

The CVPs were routinely implanted via the right subclavian vein under ultrasound guidance, as this approach does not necessitate an incision in the neck or a subcutaneous tunnel, which is considered advantageous for cosmetic reasons, particularly in younger patients. In cases where puncture of the right subclavian vein failed, the contralateral subclavian vein or internal jugular vein was selected as an alternative access site. Following the puncture of the subclavian vein, a 0.035” guidewire from the catheter kit was inserted into the inferior vena cava. A continuous incision measuring approximately 3 cm was made on the skin beginning from the puncture point. A subcutaneous pocket was created for the port via blunt dissection. Next, a peel-away sheath from the catheter kit was inserted and an indwelling catheter was advanced over the wire until the junction of the right atrium and superior vena cava. The peel-away sheath was removed, and the port connected to the catheter was implanted into the pocket. A buried suture with 4-0 PDS and steristrips was used to close the surgical incision. The location of the catheter tip and port were assessed via chest radiography following the procedure.

Study outcomes

The primary outcome was the CVP inversion rate, which was retrospectively evaluated by reviewing the electronic medical records of the patients. Secondary outcomes included technical success rate, intraoperative complications, and infectious complications. The procedure time, venous access site, and proportion of CVPs implanted outside the ribcage were also evaluated. Technical success was defined as the completion of CVP placement, regardless of any venous access. Intraoperative complications were evaluated according to the classification of adverse events published by the Society of Interventional Radiology [18]. CVP inversion and infectious complication rates are expressed as frequencies, percentages, and relative incidences (per 1,000 catheter-days). The CVPs implanted outside the ribcage were identified via post-procedural chest radiography and defined as >50% of the CVP body located outside the ribs.

Statistical analysis

Sample characteristics are reported as the number of observations and percentages for categorical variables and as the median and range for continuous ones. Fisher’s exact test was used, where appropriate, to assess categorical outcome measures. The Student’s t-test and Mann-Whitney U test were used to compare parametric and non-parametric continuous variables, respectively. P-values <0.05 were considered statistically significant. All analyses were performed using EZR software [19] (version 1.61; Saitama Medical Center, Jichi Medical University, Saitama, Japan), which is a graphical user interface for R (The R Foundation for Statistical Computing, Vienna, Austria).

## Results

Patients

**Figure 2:**
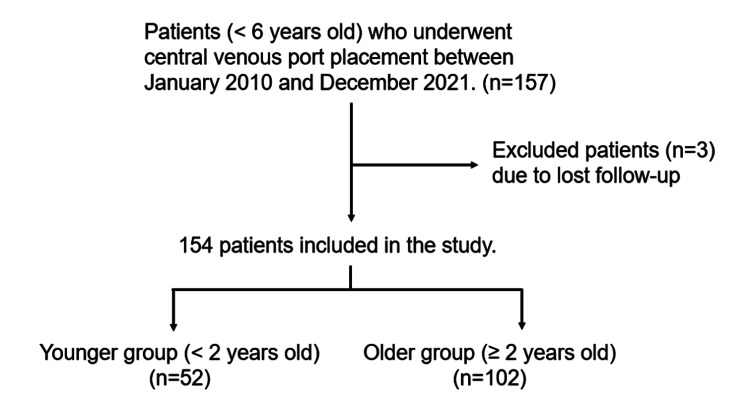
Flowchart of the study population.

We identified 157 consecutive children aged under six years who underwent CVP placement at our center between January 2010 and December 2021. Of these, 154 children (75 boys; median age, 28.5 months; range, 2-71 months) were included after excluding three patients who were lost to follow-up. The indication for CVP placement in this population was for the administration of chemotherapy, and most patients had been diagnosed with retinoblastoma (65.6%). The children were stratified based on age, with those under two years old classified as the younger group and those aged ≥two years old classified as the older group, under the assumption that younger children may have a higher risk of port inversion (Figure 2). Although the older group had significantly higher median height and weight values, there was no significant difference in terms of the Kaup index, which is commonly used in place of body mass index as an indicator of obesity in children [20]. It is defined as weight (g) divided by the square of the height (cm^2^), multiplied by a factor of 10. The proportion of low platelet count (<50,000/mm^3^) was significantly higher in the older patient group. The demographic and clinical characteristics of the patients are summarized in Table 1.

**Table 1:** Patient characteristics.

	All patients (n = 154)	Younger group (<2 years old) (n = 52)	Older group (≥2 years old) (n = 102)
Age at procedure (months)	-		
Median (range)	28.5 (2-71)	9 (2–23)	45 (24–71)
Sex (% male)	49.0	33.9	56.8
Height (median cm, range)	89 (42-116)	72 (42–85)	97 (79–116)
Weight (median kg, range)	12.3 (5.8-22.1)	9 (5.8–13)	14.2 (9.2–22.1)
Kaup index	15.9 (12.2-39.7)	17.1 (12.2–39.7)	15.4 (12.5–18)
Diagnosis	-		
Retinoblastoma	102 (66.2%)	52 (100%)	50 (49.0%)
Neuroblastoma	17 (11.0%)	-	17 (16.7%)
Leukemia/Lymphoma	15 (9.7%)	-	15 (14.7%)
Rhabdomyosarcoma	10 (6.5%)	-	10 (9.8%)
Others	10 (6.5%)	-	10 (9.8%)
Antibiotic prophylaxis	29 (18.8%)	8 (15.4%)	21 (20.6%)
White blood cell count	-		
<1,000 mm^-3^	2 (1.3%)	0 (0%)	2 (2.0%)
≥1,000 mm^-3^	152 (98.7%)	52 (100%)	100 (98.0%)
Platelet count	-		
<50,000 mm^-3^	8 (5.2%)	0 (0%)	8 (7.8%)
≥50,000 mm^-3^	146 (94.8%)	52 (100%)	94 (92.2%)

Procedural outcomes

Overall, seven (4.6%) patients experienced port inversion at a median time of 23 days (range = 2-87 days) from insertion, equivalent to 0.08/1,000 catheter-days. Although we initially attempted manual de-reversal, all cases required removal and replacement of the port with a new one. The new ports were replaced by puncturing the contralateral subclavian vein in all cases. The port inversion rate was significantly higher in the younger group than in the older group (younger group, n = 6 (11.8%); older group, n = 1 (1.0%); p = 0.00623). This significant difference showed the same trend for the frequency per 1,000 catheter-days (younger group, 0.19/1,000 catheter-days; older group, 0.02/1,000 catheter-days). The CVP was successfully implanted in 153 (99.4%) patients. Implantation failed in one patient because of laryngospasm during the procedure.

Three (1.9%) mild adverse events, including a small pneumothorax (n = 1, 0.6%), accidental puncture of the subclavian artery (n = 1, 0.6%), and laryngospasm (n = 1, 0.6%), were observed during the procedure. No moderate or severe adverse events were observed concerning CVP placement. There was no significant difference in the proportion of adverse events between the two groups (younger group, n = 1 (1.9%); older group, n = 2 (2.0%)). Infectious complications were observed in 16 (10.5%) patients at a median time of 72 days from insertion, equivalent to 0.19/1,000 catheter-days. This rate did not differ significantly between the two groups (younger group, n = 9 (17.6%); older group, n = 8 (7.8%)).

The median procedure time was 30 minutes. CVP implantation was performed through the right subclavian, left subclavian, and right jugular veins in 120 (78.4%), 22 (14.4%), and 11 (7.2%) patients, respectively. The puncture site was changed to the alternative point in 28 (18.3%) patients due to difficulty in puncturing at a similar rate in both groups (younger group, n = 8 (15.7%); older group, n = 20 (19.6%)). The proportion of CVPs implanted outside the ribcage was significantly higher in the younger group than in the older group (under two years old, 43.1%; ≥two years old, 10.8%; p = 0.00000167).

The details of the implanted CVP products were as follows: in the younger group, the 5 Fr CELSITE BABY PORT (Toray Medical, Tokyo, Japan) was used in 45 (88.2%) patients, the 5 Fr CELSITE SMALL PORT was used in five (9.8%) patients, and one (2.0%) patient was implanted with the 6 Fr Orphis CVP NEO (Sumitomo Bakelite Co., Tokyo, Japan). In the older group, the 5 Fr CELSITE SMALL PORT was used in 42 (41.2%) patients, the 5 Fr CELSITE BABY PORT in 47 (46.1%) patients, the 6 Fr CELSITE SMALL PORT in eight (7.8%) patients, the 5 Fr ORCA Baby port (Sumitomo Bakelite Co., Tokyo, Japan) in three (2.9%) patients, and the 6 Fr Orphis CVP NEO in two (2.0%) patients. The procedural outcomes are summarized in Table 2.

**Table 2:** Procedural outcomes.

	Younger group (<2 years old) (n = 52)	Older group (≥2 years old) (n = 102)	P-value
Technical success	51 (98.1%)	102 (100%)	-
Procedure time (minute)	-		
Median (range)	37 (20–105)	30 (15–75)	-
Venous access site	-		
Right subclavian vein	41 (80.4%)	79 (77.5%)	-
Left subclavian vein	6 (11.8%)	16 (15.7%)	-
Right jugular vein	4 (7.8%)	7 (6.9%)	-
Conversion to an alternate site	8 (15.7%)	20 (19.6%)	-
CVP location	-		
Outside the ribcage	22 (43.1%)	11 (10.8%)	-
Total catheter-days	-		
Median (range)	483 (4–1993)	497 (35–2609)	-
Complications	-		
Port inversion	6 (11.8%)	1 (1.0%)	0.00623
Incidence/1,000 catheter-days	0.19	0.02	-
Mean time to port inversions (days)	23.5	11	-
Infectious complications	9 (17.6%)	8 (7.8%)	-
Incidence/1,000 catheter-days	0.29	0.16	-
Mean time to infection (days)	65	100.5	-

Factors affecting central venous port inversion

The results of the univariate analysis are presented in Table 3. The univariate analysis indicated that the CVP inversion rate was significantly higher in the younger group than in the older group (p = 0.00576). No significant between-group differences were observed in terms of sex, Kaup index, port model, or operator experience (p > 0.05). Other factors that were more common in the CVP inversion group included venous access site (jugular vein) (p = 0.095) and CVP location (external to the ribcage) (p = 0.148).

**Table 3:** Univariate analysis of factors affecting central venous port (CVP) inversion.

Variable	Univariate analysis			
	Variable	CVP inversion	No inversion	P-value
		n (%)	n (%)	
Sex	Male	4	71	0.716
	Female	3	75	-
Age	<2 years old	6	45	0.00576
	≥2 years old	1	101	-
Kaup index		15.97 ± 2.46	16.18 ± 2.95	0.876
Venous access site	Subclavian vein	5	136	0.095
	Jugular vein	2	10	-
CVP location	External to the ribcage	3	28	0.148
	Internal to the ribcage	4	118	-
Implanted port	CELSITE BABY	5	83	1
	CELSITE SMALL	2	50	-
Operator’s experience	Board-certified physician	3	76	0.713
	Trainee	4	70	-

## Discussion

We assessed CVP inversion rates and explored their associated risk factors in pediatric patients and found that the CVP inversion rate in children under six years of age was 4.6% (n = 7/153), equivalent to 0.08/1,000 catheter-days. Moreover, our study showed that younger children (under two years old) had an increased risk of port inversion than older ones (≥two years old) according to the univariate analysis (p = 0.00576). Although the inversion rate in our study was higher than those reported previously, at 0.2-2.7% [16,17,21,22], the inversion ratio in our cohort of older children (≥two years old) was 1%, which is consistent with the results of previous studies. Additionally, our investigation demonstrated that the technical success rate and intraprocedural adverse events associated with CVP placement in preschool children were 99.4% and 1.9%, respectively. These results align with previous studies which reported technical success and intraprocedural complication rates ranging 97.5-100% and 1.4-2.4%, respectively [23-25].

Limited studies have evaluated the CVP inversion rate in infants and toddlers (under two years old). Acord et al. reported only one (1.6%) inversion that occurred 57 days after placement in their retrospective study of 64 ports in infants [9]. The key difference between their study and the present investigation was the site of central venous catheterization. Acord et al. implanted catheters through the internal jugular vein, whereas the subclavian vein was the primary access site in our study. Considering the puncture site of the subclavian vein, the CVP was implanted closer to the shoulder joint than that performed via the internal jugular vein approach. Moreover, younger children experience frequent rotational movements of the shoulders (such as during crawling), which may increase the likelihood of CVPs near the shoulder joint moving readily within the pocket and potentially flipping over [26]. In addition, based on the guidelines provided by the American Academy of Pediatrics, babies start waking between 9 and 18 months, which means the younger group (under two years old) will exhibit a higher frequency of crawling compared to the older group (≥two years old) [27].

Therefore, we hypothesized that this patient demographic would experience a higher rate of flipping than previously reported. For these reasons, we also hypothesized that the subclavian vein approach might represent a risk factor for port inversion; however, our study did not find any statistical significance for this factor (CVP inversion group, 71.4%; no inversion group, 93.1%; p = 0.095). Similarly, we hypothesized that CVPs placed external to the ribcage may have a higher inversion rate for similar reasons but found no statistical significance (CVP inversion group, 42.9%; no inversion group, 19.2%; p = 0.148). The smaller body size of the younger group, which exhibited a high rate of CVP inversion, may have resulted in a higher likelihood of CVP implantation external to the ribcage.

According to the previous reports [21,28], proposed risk factors for port inversion in adults include loose or excessive subcutaneous tissue and oversized pockets, which permit excessive port movement. Although port fixation using non-absorbable sutures is recommended by the manufacturer in the instructions for use to prevent port inversion, McNulty et al. [21] claimed that suture fixation is not routinely necessary and may negatively impact port removal. This is particularly true for young children with thick subcutaneous fat, where it is often difficult to secure the port firmly to the pectoralis muscle, suggesting that port fixation may play a smaller role than in adults. To prevent port inversion, we suppose it is crucial to create the smallest possible pocket that accommodates the port.

Twiddler’s syndrome, which is similar to CVP inversion, is a widely recognized complication in cardiology. It is characterized by the failure of a permanent pacemaker due to the patient’s conscious or unconscious rotation of the pacemaker’s pulse generator within the subcutaneous pocket, which ultimately leads to lead displacement [29]. Excessive subcutaneous tissue has been identified as a risk factor for Twiddler’s syndrome because it allows the device to rotate freely within the pocket [30-32]. The adiposity of human newborns peaks during early infancy and then undergoes a drastic decline, leading to a relatively lean state by the age of five years. Consequently, younger children (under two years old) tend to have more adipose tissue than older individuals. It may potentially increase the likelihood of Twiddler’s syndrome and CVP inversion in this population [33]. However, it is unclear whether patient manipulation of CVPs contributed to inversion in our study, as there was no evidence of intentional rotation in the patients’ medical records.

Our study has some limitations. First, this study was limited by its retrospective nature. Retrospective analyses have inherent selection bias and the possibility of missing data. In this case, selection bias was reduced by analyzing consecutive patients implanted with CVPs over a period of 12 years. Second, the involvement of multiple radiologists in the CVP placement process. Although our study indicated that the CVP inversion rate did not vary based on whether the operator was a board-certified physician or not, discrepancies in each operator’s preferences may have influenced the CVP inversion rate through factors such as the selected needle, the specific CVP product, the suture fixation of the ports into the pockets, and the pocket sizes, all of which depend on the discretion of the attending radiologist. Although the previous report has claimed that suture fixation does not influence port inversion [21], the presence or absence of suture fixation may have influenced the occurrence of port inversion. Third, the assessment of CVP inversion based on electronic medical records may have led to an underestimation of the actual incidence of CVP inversion. Finally, the small sample size of the CVP inversion group was also a limitation, as it may have resulted in inadequate statistical power for the analysis.

## Conclusions

The CVP inversion rate in children under six years of age was 4.6%. Younger children (under two years old) may be at an elevated risk of port inversion compared to older ones (≥two years old).
